# LINC00518: a key player in tumor progression and clinical outcomes

**DOI:** 10.3389/fimmu.2024.1419576

**Published:** 2024-07-23

**Authors:** Qiang Yi, Gangfeng Zhu, Weijian Zhu, Jiaqi Wang, Xinting Ouyang, Kuan Yang, Jinghua Zhong

**Affiliations:** ^1^ The First Clinical Medical College, Gannan Medical University, Ganzhou, Jiangxi, China; ^2^ Department of Oncology, The First Affiliated Hospital of Gannan Medical University, Ganzhou, Jiangxi, China

**Keywords:** lncRNA, LINC00518, tumor, expression, mechanism, biological marker

## Abstract

Long non-coding RNAs (lncRNAs), defined as RNA molecules exceeding 200 nucleotides in length, have been implicated in the regulation of various biological processes and the progression of tumors. Among them, LINC00518, a recently identified lncRNA encoded by a gene located on chromosome 6p24.3, consists of three exons and is predicted to positively regulate the expression of specific genes. LINC00518 has emerged as a key oncogenic lncRNA in multiple cancer types. It exerts its tumor-promoting effects by modulating the expression of several target genes, primarily through acting as a sponge for microRNAs (miRNAs). Additionally, LINC00518 influences critical signaling pathways, including the Wnt/β-catenin, JAK/STAT, and integrin β3/FAK pathways. Elevated levels of LINC00518 in tumor tissues are associated with increased tumor size, advanced clinical stage, metastasis, and poor survival prognosis. This review provides a comprehensive summary of the genetic characteristics, expression patterns, biological functions, and underlying mechanisms of LINC00518 in human diseases.

## 1 Introduction

Cancer is one of the leading causes of death worldwide, with over 10 million people succumbing to it each year, posing a significant threat to human life (1). Studies indicate that the future burden of cancer is immeasurable, making it imperative to curb the persistent burden of cancer (2). Despite the development of various strategies for cancer treatment in recent years, including surgery, radiotherapy, and chemotherapy, the efficacy of treatments often remains limited (3). Targeted therapies have emerged as crucial in cancer treatment, yielding notable advancements; however, they have not significantly improved the overall survival rates of cancer patients (4). Consequently, there is a need to develop new targets and biomarkers for diagnosing and predicting cancer.

Long non-coding RNA (lncRNA) is defined as a group of RNA molecules exceeding 200 nucleotides in length, lacking protein-coding functions (5). In cancer, lncRNAs regulate tumor cell cycle, proliferation, migration, and therapeutic resistance by interacting with DNA, mRNA, proteins, and miRNAs, thereby promoting adverse outcomes in malignant tumors (6). For instance, lncRNA-CBSLR forms a CBSLR/YTHDF2/CBS complex by recruiting the YTHDF2 protein and CBS mRNA. This complex promotes m6A-YTHDF2-dependent regulation of CBS, thereby modulating ferroptosis in gastric cancer, which in turn influences treatment resistance and contributes to poor prognosis in solid tumors (7). Furthermore, the transcription levels of lncRNAs are regulated by transcription factors and DNA methylation (8). These studies collectively unveil the potential of lncRNAs as clinical biomarkers and therapeutic targets in various cancers, providing insights into their multifaceted roles in cancer biology.

*LINC00518* (ENSG00000183674), also known as *LENOX* or *C6orf218*, was first discovered in 2014 and has since emerged as a highly valuable non-invasive method for distinguishing melanoma from nevi. It also serves as a candidate gene contributing to social disinhibition in Mexican American youth (9, 10). Since its initial discovery, extensive research has reported that LINC00518 can regulate various cancer-related phenomena, including cell cycle regulation, proliferation, migration, invasion, epithelial-mesenchymal transition (EMT), radioresistance, and drug resistance. LINC00518 achieves these regulatory functions by modulating signaling pathways such as Wnt/β-catenin, JAK/STAT3, and integrin β3/FAK.

This review aims to elucidate the genetic structure, abnormal expression levels, biological functions, associated mechanisms, and potential clinical applications of LINC00518 in human diseases. It also summarizes its role as a biomarker for diagnosis, prediction, and treatment of human diseases.

## 2 Genetic information for LINC00518

**Figure 1:**
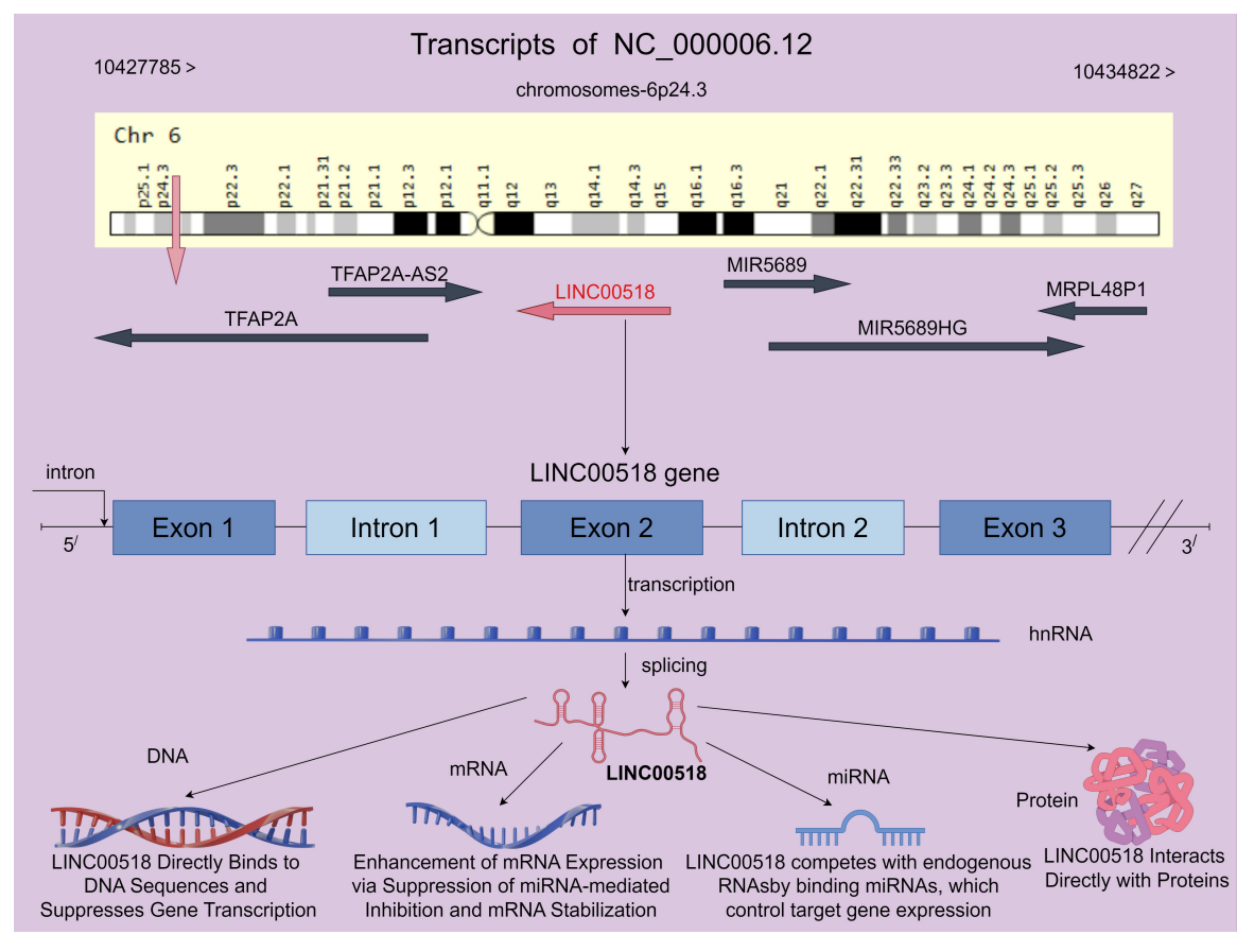
LINC00518 is located on human chromosome 6 and comprises 3 exons, with the potential to generate up to 8 transcript variants according to the NCBI database. Furthermore, LINC00518 demonstrates diverse functions across multiple biological levels, including DNA, mRNA, miRNA, and protein interactions. These multifaceted roles collectively regulate the biological functions of LINC00518 in tumors (the chromosome map is provided by the Genecard website, while the rest is created using the Figdraw 2.0 platform).

LINC00518 gene is transcribed from chromosome 6p24.3, spanning 7,038 nucleotides across the genomic DNA. It is located between the TFAP2A and GCNT2 genes, with distances of 8,126 bp and 86,529 bp, respectively. In the latest assembly GRCh38, LINC00518 (NC_000006.12) is positioned on chromosome 6, from nucleotide 10,427,785 to 10,434,822, totaling 7,038 nucleotides (as shown in **Figure 1**). LINC00518 comprises 3 exons and 2 introns, with 8 transcripts predicted to positively influence the expression of certain genes (https://www.ncbi.nlm.nih.gov/gene/221718).

## 3 Clinical sample study of LINC00518

Long non-coding RNAs (lncRNAs) are a class of transcripts that do not encode proteins and have gained increasing attention. As shown in the **Table 1**, LINC00518 exhibits significant dysregulation in expression levels in various diseases such as cervical cancer, head and neck squamous cell carcinoma, colorectal cancer, and breast cancer, and is associated with multiple clinical features.

**Table 1:** The Clinicopathological characteristics of LINC00518 in various malignancies.

Cancer type	Number of cases	Expression	Clinicopathological characteristics	Prognosis	Refs.
Cervical cancer (CC)	133	high	FIGO stage, lymph node metastasis and depth of cervical invasion	poor	(11)
Breast cancer (BC)	30	high	Grade, Tumor stage (T)	poor	(12)
Lung Adenocarcinoma (LUAD)	42	high	Tumor size, TNM stage	poor	(13)
Melanoma	40	high	poor overall survival	poor	(14)
	36	high	clinical stage, poor overall survival	poor	(15)
Uveal Melanoma	41	high	/	poor	(16)
Colorectal cancer (CRC)	30	high	poor overall survival	poor	(17)
Multiple Myeloma	43	high	/	poor	(18)
Prostate cancer (PC)	55	high	relatively poor outcome	poor	(19)

### 3.1 Breast cancer

LINC00518 is notably overexpressed in breast cancer tissues (12). Interestingly, the mRNA expression levels of multidrug resistance protein 1 (MRP1) are positively correlated with those of LINC00518 in these tissues. This correlation implies a potential role of LINC00518 in the regulation of MRP1 expression, which is a critical factor in the development of multidrug resistance in cancer (20). Therefore, targeting LINC00518 may offer a novel therapeutic approach for overcoming multidrug resistance in breast cancer.

### 3.2 Melanoma

Reportedly, LINC00518 is significantly overexpressed in melanoma tissues compared to adjacent normal tissues. According to Kaplan-Meier analysis, the overall survival period is longer in the low LINC00518 expression group compared to the high expression group (14). Notably, high levels of LINC00518 are associated with advanced clinical staging of melanoma, indicating its potential role in the progression of the disease. Furthermore, univariate and multivariate Cox regression analysis further confirms that high expression of LINC00518 is an independent risk factor for melanoma patients (15)​. In clinical application, LINC00518, as a key gene in pigment lesion analysis (PLA), has been used to exclude melanoma (21–27). This dual-gene PLA exhibits high repeatability and reproducibility (28–30). Moreover, the genetic analysis efficiency of LINC00518 and PRAME is significantly higher than that of TERT (TERT is commonly used as a marker to identify malignant melanoma) (31). Wan Q et al. (32) conducted an in-depth study on the regulation of specific lncRNAs in melanoma and their correlation with tumor-infiltrating macrophages. They constructed a more accurate and effective LINC00518-related model, offering a simpler and more reliable prediction method for melanoma patients, and establishing a framework for evaluating potential candidates for immunotherapy. This paves the way for future individualized immunotherapy approaches in melanoma treatment. Additionally, studies have shown significant overexpression of LINC00518 in vitreous fluid, serum, and vitreous and serum extracellular vesicles in uveal melanoma tissues (16). Therefore, LINC00518 can be considered a biomarker for early cancer detection.

### 3.3 Lung adenocarcinoma

Clinical data reveals that the expression of LINC00518 is significantly elevated in LUAD tumor tissues compared to normal lung tissues. Furthermore, data from the TCGA dataset indicates that patients with high expression of LINC00518 have a poorer overall survival rate. It is noteworthy that LINC00518 shows significant correlations with tumor size and TNM staging. Additionally, Hu J et al. (33) identified characteristics of LINC00518 as candidate prognostic biomarkers for predicting OS in LUAD patients. Subsequently, Wu X et al. (34) proposed a novel lncRNA-miRNA-mRNA ceRNA network related to LINC00518 in LUAD, highlighting its indispensable role in tumors. These studies further support the potential of LINC00518 in cancer diagnosis.

### 3.4 Multiple myeloma

In multiple myeloma (MM), LINC00518 is overexpressed in MM samples (MM cells LP1 and KMS11) compared to normal samples and human bone marrow cells (HS-27A). Further studies showed that the relative expression level of LINC00518 differed in sensitive (LP1 and KMS11) and drug resistant (LP1 and KMS11) MM cells cultured in media, with significantly elevated levels in the resistant cells (18). Based on this evidence, we speculate that LINC00518 may function as an oncogene in the process of multiple myeloma development. However, the functional role of LINC00518 needs validation in large-scale clinical cohorts.

### 3.5 Other malignant tumors

In cervical cancer, the overexpression of LINC00518 is significantly associated with worse International Federation of Gynecology and Obstetrics (FIGO) staging, lymph node metastasis, greater depth of cervical infiltration, and lower overall survival in cervical cancer patients (11). In head and neck squamous cell carcinoma, the expression level of LINC00518 is significantly negatively correlated with the average methylation level. Low DNA methylation promotes high expression of LINC00518, thereby increasing patient pathological grading and staging (35). Additionally, Wang X et al. (36) studied the LINC00518-related ceRNA network in NSCLC, which can distinguish between normal and cancerous tissues. Other studies have indicated that LINC00518 promotes tumorigenesis in colorectal cancer and prostate cancer (17, 19).

## 4 Animal studies of LINC00518

The upregulation of LINC00518 has been shown to promote the metastasis and *in vivo* growth of breast cancer. Conversely, downregulation of LINC00518 has been reported to have the opposite effect in lung adenocarcinoma, non-small cell lung cancer, and malignant melanoma of the skin (as shown in the **Table 2**).

**Table 2:** Effects of LINC00518 on growth and metastasis of cancer xenografts.

Cancer type	Animal models	Function	References
Breast cancer	Xenograft model of breast cancer (nude mice),the MCE-7 cells transfected with oe-LINC00518 and sh-LINC00518 were inoculated	↑↑ LINC00518: ↑ tumor growth	(37)
Lung adenocarcinoma	Xenograft model of LUAD (nude mice), injection LINC00518 knockdown cells	↓↓LINC00518: ↓tumor growth	(38)
	Inoculating A549 cells transfected with si-LINC00518 into nude mice	↓↓LINC00518:↓tumor growth, ↓the expression of Ki-67 and p-AKT	(13)
Non-small cell lung cancer	Xenograft model of NSCLC (nude mice) in the si-LINC00518	↓↓LINC00518:↓tumor growth, ↓the expression of Ki67, PCNA, MMP7 and MMP9	(39)
cutaneous malignant melanoma	Repression of LINC00518 expression using an shLINC00518 plasmid, or Santacruzamate A(xenograft mouse model)	↓↓LINC00518: ↓tumor growth, ↑radiosensitivity in the subcutaneous sarcoma model	(40)
	Stably expressing LINC00518 shRNA or miR-204–5p A375 cells were tail vein injected into nude mice	↓↓LINC00518:↓the number of metastatic lung nodules	(15)

↑↑ LINC00518: ↑↑ means LINC00518 overexpression; ↑ : ↑ means promoting. ↓↓ LINC00518: ↓↓ means LINC00518 knockdown or knockout; ↓ : ↓ means inhibiting.

Animal studies are consistent with *in vitro* research, confirming the oncogenic role of LINC00518. In lung cancer animal models, silencing LINC00518 results in reduced tumor volume and weight. Similarly, experiments in breast cancer animal models also demonstrate the role of LINC00518 in promoting tumor growth. Furthermore, LINC00518 not only promotes tumor growth in melanoma but also increases the number of lung metastatic nodules. Therefore, the occurrence and development of most cancer types are closely related to LINC00518.

## 5 Biological role of LINC00518 in cancer

**Figure 2:**
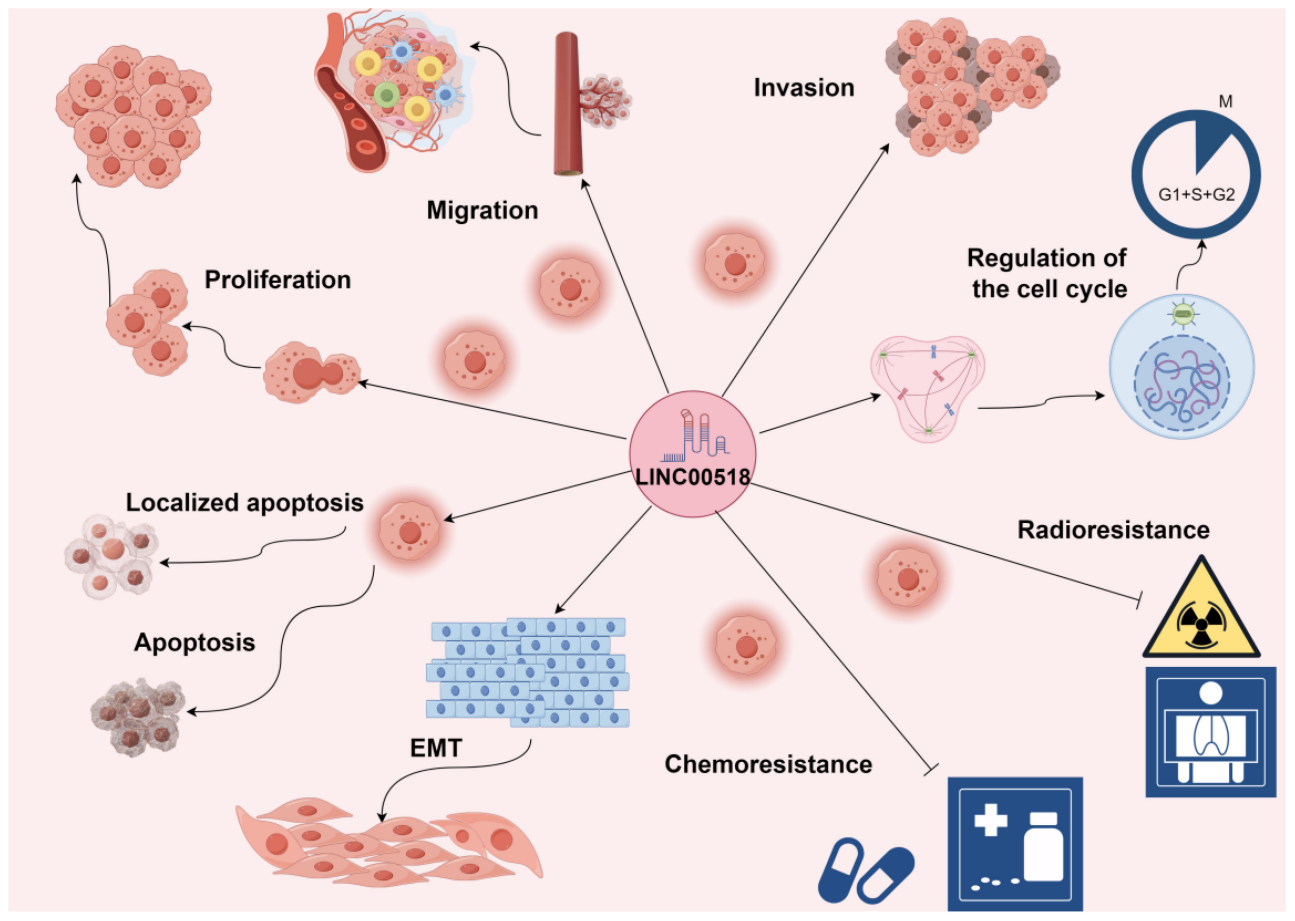
LINC00518 plays a crucial role in the initiation and progression of tumors. Its overexpression significantly promotes tumor cell proliferation, invasion, migration, epithelial-mesenchymal transition (EMT), and tumor angiogenesis, while also reducing tumor cell apoptosis. Additionally, LINC00518 has been observed to decrease drug sensitivity during radiotherapy and chemotherapy, thereby impacting treatment efficacy. Promotes →; Inhibits ⟞.

LINC00518 is pivotal in tumor initiation and progression. Its overexpression enhances tumor cell proliferation, invasion, migration, and epithelial-mesenchymal transition (EMT), while reducing apoptosis. Additionally, LINC00518 decreases drug sensitivity during radiotherapy and chemotherapy, affecting treatment efficacy (As shown in **Table 3** and **Figure 2**). These roles underscore LINC00518’s potential as a therapeutic target in cancer.

**Table 3:** Biological role of LINC00518 in various cancers.

Cancer	Expression	Functional	Related gene	Role	Reference
LUAD	Upregulated	proliferation, cell cycle	miR-185–3p/MECP2	oncogene	(13)
NSCLC	Upregulated	proliferation, invasion, and migration	miR-216b-5p	oncogene	(39)
	Upregulated	proliferation,migration,cell cycle	miR-335–3p/CTHRC1	oncogene	(38)
Melanoma	Upregulated	proliferation, invasion, and migration	miR-526b-3p/EIF5A2	oncogene	(14)
	Upregulated	radioresistance	miR-33a-3p/HIF-1α	oncogene	(40)
	Upregulated	invasion and migration	miR-204–5p/AP1S2	oncogene	(15)
Uveal Melanoma	Upregulated	metastasis-related processes	/	oncogene	(16)
CC	Upregulated	proliferation, migration and invasion, EMT, and induced apoptosis	JAK/STAT3	oncogene	(11)
BC	Upregulated	MDR	miR-199a/MRP1	oncogene	(12)
	Upregulated	proliferation, invasion, migration, EMT while enhancing apoptosis.	CDX2/Wnt	oncogene	(37)
	Upregulated	cancer stemness and tumorigenesis	miR-185–3p/E2F1/Nanog	oncogene	(41)
PC	Upregulated	paclitaxel resistance	miR-216b-5p/GATA6	oncogene	(19)
MM	Upregulated	resistance to melphalan chemotherapy	miR-140–5p/ATG14	oncogene	(18)

### 5.1 LINC00518 influences the cell cycle

LINC00518 has been shown to control the cell cycle during tumor development. For instance, Han X et al. (13) demonstrated that downregulation of LINC00518 led to an increase in the proportion of cells in the G1 phase and a decrease in the proportion of cells in the S phase in A549 and PC9 cells. These results suggest that the expression of LINC00518 accelerates the proliferation of LUAD cells by prolonging the S phase and reducing the G1 phase proportion. Another study by Shen R et al. (38) found that knocking down LINC00518 resulted in a significantly lower proportion of cells in the S phase and a higher proportion of cells in the G0/G1 phase in LUAD cells. In summary, overexpression of LINC00518 can regulate the cell cycle, leading to adverse outcomes for patients.

### 5.2 LINC00518 regulates tumor cell proliferation and apoptosis

Functional biology analysis indicates that LINC00518 promotes cervical cancer cell proliferation and inhibits apoptosis (11). Knockdown of LINC00518 significantly impairs the proliferation of SiHa and HeLa cells and increases apoptosis. Wang HB et al. (37) demonstrated that silencing LINC00518 promotes apoptosis in breast cancer epithelial cells, inhibits cell growth and proliferation, reduces colony formation ability, and inhibits invasion and migration capabilities. Furthermore, Han X et al. (13) showed that inhibiting the expression of LINC00518 significantly reduces the protein levels of proliferation-related proteins (p-AKT, p-ERK, and cyclin D1) in LUAD cells, with the opposite results observed after overexpressing LINC00518. Recently, it was found that interference with LINC00518 leads to a decrease in the clonogenicity of NSCLC cells and inhibits tumor cell proliferation (39). In melanoma, knockdown of LINC00518 significantly inhibits the proliferation of A375 and A2058 cells (14). Additionally, Liu Y et al. (40) found that compared to the control group, cells with silenced LINC00518 show significantly reduced proliferation and increased apoptosis, with a significant decrease in cell survival. Overall, LINC00518 is a significant contributor to the process of tumor formation. However, its other pan-cancer biological functions require further investigation.

### 5.3 LINC00518 promotes tumor cell migration and invasion

Cell invasion and migration are crucial events that can transform malignant tumors from local growth to metastatic colonization, which is associated with a higher risk of mortality (42). With advancements in novel biotechnologies and experimental tools, research on the mechanisms of metastasis has increased, revealing the role of lncRNAs in this fundamental capability of different cancer cells. Wang DW et al. (11) found that LINC00518 plays a critical role in regulating the migration and invasion of cervical cancer (CC) cells, and it can inhibit CC metastasis by influencing the epithelial-mesenchymal transition (EMT) pathway. Further studies have shown that interference with LINC00518 can suppress the migration and invasion of breast cancer and non-small cell lung cancer (37, 39). Similarly, in melanoma, silencing LINC00518 has an inhibitory effect on the invasion, migration, and infiltration of melanoma cells, as demonstrated by lower migration and invasion capabilities in cutaneous malignant melanoma (CMM) cells with silenced LINC00518 compared to control cells (14, 15, 40). However, interestingly, Barbagallo C et al. (16) found that LINC00518 may be involved in metastasis-related processes rather than cell proliferation and is not controlled by cell cycle progression. In summary, LINC00518 plays a significant role in enhancing cancer cell motility and promoting cancer metastasis.

### 5.4 LINC00518 facilitates the process of epithelial-mesenchymal transition

The epithelial-mesenchymal transition (EMT) delineates the acquisition of mesenchymal traits by epithelial cells, a pivotal process tightly entwined with tumor initiation, invasion, metastasis, and therapeutic resistance in cancers (43). Notably, long non-coding RNAs (lncRNAs) such as HOTAIR, MALAT1, LincRNA-RoR, and H19 emerge as prominent regulators orchestrating the EMT cascade (44). Specifically, silencing LINC00518 in cervical cancer cells leads to a significant reduction in N-cadherin and vimentin expression while elevating the epithelial marker E-cadherin’s protein levels (11). Likewise, in breast cancer, downregulation of LINC00518 impedes Wnt signaling activation, thereby restraining the expression of EMT-associated proteins Slug, Snail1, Twist1, ZEB1, and ZEB2, while promoting E-cadherin expression (37). In essence, the intricate interplay between LINC00518 and the genetic attributes of EMT in cancer unveils a compelling nexus.

### 5.5 LINC00518 promotes tumor cell therapy resistance

Therapeutic resistance stands as a pivotal determinant of cancer mortality rates (45). Studies reveal that LINC00518, through sequestering miR-199a, augments MRP1 expression, thereby enhancing multidrug resistance to ADR (Adriamycin), VCR (Vincristine), and PTX (Paclitaxel) in breast cancer cells, inhibiting apoptosis, and reducing caspase 9 activity (12). Additionally, in prostate cancer, LINC00518 exhibits significantly higher relative levels in acquired paclitaxel-resistant prostate cancer cells, indicating its pivotal role in paclitaxel resistance (19). Further investigations highlight LINC00518 overexpression in melphalan-resistant cells, while its knockdown diminishes melphalan resistance in multiple myeloma cells. LINC00518 promotes autophagy in multiple myeloma cells, consequently bolstering resistance to melphalan chemotherapy (18). Although animal experiments are lacking to fully elucidate LINC00518’s role in promoting chemotherapy resistance in tumor cells, clinical trials bridge this gap. Collectively, these findings underscore the significant involvement of LINC00518 in tumor chemoresistance. Moreover, as chemotherapy constitutes one of the three pillars of cancer treatment, LINC00518’s role in radioresistance has also been explored. Radiotherapy (RT) (46), a modality in cancer treatment either standalone or in conjunction with surgery, chemotherapy, immunotherapy, and targeted therapy, has been investigated in relation to LINC00518 by Liu et al. (40), revealing its promotion of glycolytic metabolism, thereby increasing radioresistance in CMM cells, resulting in suboptimal radiotherapy outcomes and poorer prognosis for patients. In conclusion, overcoming the challenges posed by LINC00518-mediated treatment resistance heralds new hope for patients.

## 6 Mechanisms of LINC00518-mediated biological functions in cancer

**Figure 3:**
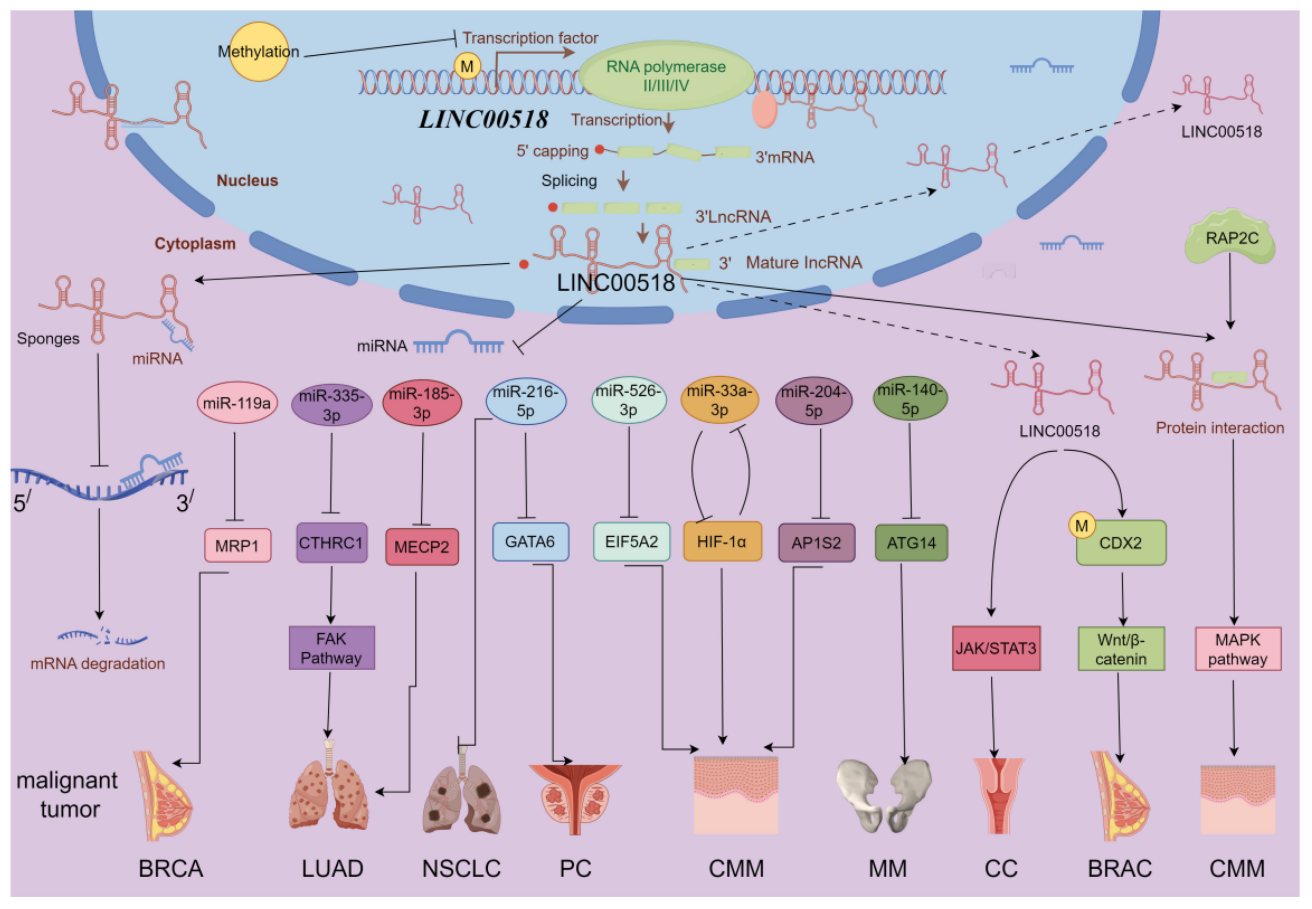
LINC00518-related gene network and potential downstream regulatory mechanisms (by Figdraw 2.0). BRCA, breast cancer; PC, prostate cancer; NSCLC, non-small cell lung cancer; LUAD, lung adenocarcinoma; CMM, melanoma; MM, multiple myeloma; CC, clear cell renal cell carcinoma. Promotes →; Inhibits ⟞.

LINC00518 influences cancer through multiple mechanisms, including lncRNA-miRNA-mRNA ceRNA networks and activation of JAK/STAT3, Wnt/β-catenin, Integrin β3/FAK, and MAPK signaling pathways. It also interacts with HIF-1α, contributing to cancer progression (As in **Figure 3**). These interactions highlight LINC00518’s complex role in tumor biology.

### 6.1 lncRNA-miRNA-mRNA ceRNA networks

**Figure 4:**
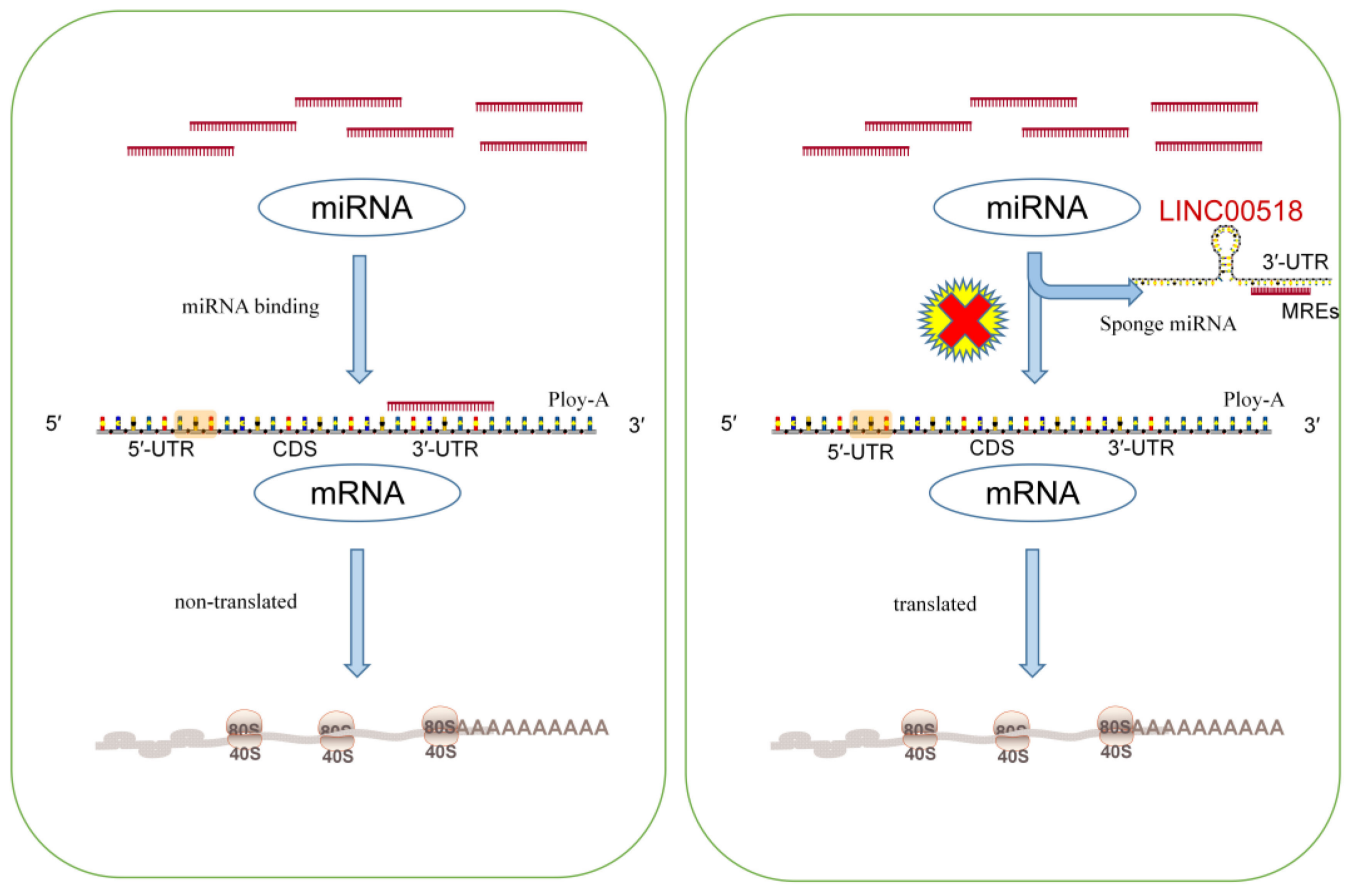
The left diagram illustrates the general process of miRNA inhibiting mRNA translation. The right figure demonstrates how lncRNA acts like a sponge to adsorb miRNA, thereby negating its original function of inhibiting mRNA translation. Consequently, by directly interacting with miRNAs, lncRNAs can indirectly regulate other downstream genes.

The competitive endogenous RNA (ceRNA) regulatory network represents a key mechanism through which LINC00518 operates in cancer development. By functioning as a miRNA sponge, LINC00518 regulates the expression of downstream messenger RNAs (mRNAs), thus influencing tumor progression (As in **Figure 4**).

In breast cancer, LINC00518 acts as a sponge for miR-199a, thereby modulating the miRNA target MRP1 and functioning as an oncogene (12). The multidrug resistance-associated protein 1 (MRP1, ABCC1), an ABC transporter protein, generates multidrug resistance (MDR) by altering cellular redox balance, rendering cells susceptible to specific drugs (47). In lung adenocarcinoma, LINC00518 suppresses miR-335–3p, leading to increased levels of CTHRC1 and promoting tumor progression. Moreover, LINC00518 enhances MECP2 expression by sponging miR-185–3P, further driving LUAD development (13, 38). Elevated CTHRC1 and MECP2 expression are associated with the occurrence, proliferation, invasion, and metastasis of various human malignancies (48, 49). In non-small cell lung cancer, LINC00518 competitively binds miR-216–5p to upregulate MMP7 and MMP9 mRNA and protein expression, thereby enhancing tumor proliferation, migration, and invasion (39). In melanoma, LINC00518 positively regulates EIF5A2 expression by directly binding miR-526–3p, suppressing melanoma (CMM) proliferation and metastasis. EIF5A2 induces epithelial-mesenchymal transition (EMT), promoting tumor formation, enhancing cancer cell growth, and increasing cancer cell motility and metastasis (50). Additionally, LINC00518 indirectly promotes AP1S2 via miR-204–5p in melanoma, playing a crucial role in disease progression (15). AP1S2 was identified as potentially pivotal in melanoma as early as 2013 (51). A noteworthy finding in melanoma is that LINC00518, by inhibiting miR-33a-3p expression and promoting its target gene hypoxia-inducible factor 1α (HIF-1α) expression, acts as an oncogene and directly regulates miR-33a-3p in a negative feedback loop, inducing radioresistance (40). Hypoxia activation is associated with aggressive phenotypes, including resistance to radiation and chemotherapy, metastasis, and poor patient prognosis, playing a critical role in cancer (52). In prostate cancer, LINC00518 modulates GATA6 expression by competitively sponging miR-216–5p. GATA6 overexpression has been demonstrated to play a significant role in various cancers, including lung cancer, digestive system tumors, breast cancer, and ovarian cancer (53). Furthermore, LINC00518’s overexpression promotes tumor growth in multiple myeloma cells by downregulating miR-140–5p expression and upregulating ATG14 expression (18). ATG14 has been found to control tumor resistance mechanisms in pancreatic cancer, gastric cancer, and ovarian cancer, playing a crucial role in treatment prognosis (54–56).

Overall, these findings underscore the multifaceted role of LINC00518 as a crucial regulator in various cancer types, highlighting its potential as a biomarker and therapeutic target. Understanding the molecular mechanisms underlying LINC00518’s interactions with miRNAs and their downstream targets can provide valuable insights into tumor progression and resistance, offering new avenues for personalized cancer treatment.

### 6.2 JAK/STAT3 signaling pathway

The JAK/STAT3 axis is a classical intracellular signaling pathway that plays a crucial role in regulating physiological processes such as cell proliferation, apoptosis, and angiogenesis (57, 58). However, its abnormal cellular signaling and regulation are closely linked to the occurrence and development of tumors. Excessive or aberrant activation of this signaling pathway may lead to uncontrolled proliferation, enhanced survival, and invasive capabilities of tumor cells, thereby promoting tumor development and metastasis (59). Therefore, a comprehensive investigation into the functional mechanisms and regulatory pathways of the JAK/STAT3 axis holds significant importance for cancer treatment and prevention. According to reports, elevated levels of LINC00518 promote cancer progression by activating the JAK/STAT3 signaling pathway. Wang et al. (11) observed significant inhibition of phosphorylated JAK2 and phosphorylated STAT3 in LINC00518-silenced SiHa cervical cancer cells, while the protein levels of JAK2 and STAT3 remained unchanged. Similarly, upon downregulation of LINC00518 expression, the protein levels of p-JAK2 and p-STAT3 decreased in HeLa cervical cancer cells as well. Therefore, we can confidently conclude that the activation of the JAK/STAT3 signaling pathway regulated by LINC00518 enhances cell growth in cervical cancer.

### 6.3 Wnt/β-catenin signaling pathway

The Wnt signaling pathway regulates development and tissue homeostasis, and its dysregulation is associated with developmental defects, cancer, and degenerative diseases (60). Aberrant Wnt/β-catenin signaling pathway promotes cancer stem cell renewal, cell proliferation, and differentiation, playing a crucial role in tumor initiation and treatment response (61, 62). Studies have found that LINC00518 is localized in the cell nucleus in breast cancer (BC) tissues. Downregulation of LINC00518 can inhibit BC progression by suppressing CDX2 methylation and the Wnt signaling pathway, thereby inhibiting cell proliferation and promoting apoptosis of BC epithelial cells (37). Overexpression of LINC00518 can enhance β-catenin, c-Myc, CyclinD1 mRNA, and protein levels, while inhibiting GSK3β mRNA and protein levels; silencing LINC00518 has the opposite effect. There is a significant correlation between the CpG island methylation level in the CDX2 gene promoter region and LINC00518 expression. Further research indicates that LINC00518’s impact on CDX2 promoter region methylation is mediated by methyltransferases. However, there is limited research on how LINC00518 influences the Wnt/β-catenin signaling pathway, and further studies are needed to investigate the effects and mechanisms of LINC00518 on this pathway in other tumors.

### 6.4 Integrin β3/FAK signaling pathway

Focal adhesion kinase (FAK) serves as both a non-receptor tyrosine kinase and an adapter protein, primarily regulating adhesion signal transduction and cell migration, and it is overexpressed and activated in various advanced solid tumors (63, 64). Integrins can alter cell behavior by recruiting and activating FAK, leading to tumor growth and metastasis (65). Recent studies have indicated a potential relationship between LINC00518 and the AKT signaling pathway in cancer. Shen et al. (38) found that LINC00518 increases CTHRC1 expression by sponging miR-335–3p, affecting integrin β3/FAK signal transduction, and promoting proliferation and metastasis in lung adenocarcinoma (LUAD). Using the FAK inhibitor VS-6063, the addition of VS-6063 can restore the migration and invasion capabilities of LUAD cells inhibited by suppressing miR-335–3p, while the FAK inhibitor can also induce CTHRC1 and p-FAK protein levels. In summary, LINC00518 promotes tumor cell proliferation, migration, invasion, and clustering by regulating the downstream miR-335–3p-CTHRC1-integrin β3/FAK pathway.

### 6.5 MAPK signaling pathway

Ras/RAF/MEK/ERK (MAPK) signaling pathway is one of the most clearly defined pathways in cancer biology, regulating various aspects of life through a widely present signal transduction pathway, and often undergoing changes in tumors (66, 67). Gambi et al. (68) found that LINC00518 interacts with RAP2C, regulating melanoma metabolism and promoting resistance to MAPK inhibition. Silencing LINC00518 can inhibit the growth of melanoma resistant cells transplanted into mouse tumors in the presence of MAPKi. Unfortunately, research on LINC00518 and MAPK has only been conducted at the biological level, and the specific mechanism remains unclear.

### 6.6 Interaction with hypoxia-inducible factor 1α

Hypoxia-inducible factor 1 alpha (HIF-1α) stands as a pivotal transcription factor in cancer progression and targeted therapies, closely linked to tumor metastasis, angiogenesis, poor patient prognosis, and resistance to cancer treatments (69, 70). With a deeper understanding of lncRNAs, their role in regulating HIF-1α under both hypoxic and normoxic conditions in cancer has garnered attention (71, 72). Studies have revealed that LINC00518 directly targets miR-33a-3p, leading to increased HIF-1α expression in CMM (cutaneous malignant melanoma) (40). Knockdown of LINC00518 or the addition of miR-33a-3p mimetics resulted in decreased levels of HIF-1α and LDHA proteins, while silencing HIF-1α in melanoma cells significantly reduced miR-33a-3p enrichment, indicating the presence of a negative feedback loop regulating tumor cell biological traits. Further investigations unveiled that upon HIF-1α knockdown, the HIF-1α-HDAC [histone deacetylase] immunoprecipitation decreased, whereas it increased with HIF-1α overexpression. The combination of HDAC with transcription factors binding to the promoter region can inhibit gene expression by accelerating histone deacetylation (73). Therefore, HIF-1α might bind to the miR-33a promoter, inhibiting miR-33a transcription by accelerating histone deacetylation of miR-33a. In summary, LINC00518 indirectly regulates HIF-1α, promoting tumor cell proliferation, migration, invasion, and colony formation.

## 7 Conclusion and future perspectives

A substantial body of evidence indicates that lncRNAs play a crucial role in the pathogenesis of human diseases (74, 75). With further research on lncRNAs in human cancers, their role in tumor initiation and progression is worth exploring and summarizing. In this review, we comprehensively review current research on the role of LINC00518 in human tumors and provide insights into its genetic structure information. Additionally, we discuss the potential impact of LINC00518 expression levels on the prognosis of humans and other organisms like mice. LINC00518 acts as an oncogene by regulating tumor cell cycle, proliferation, apoptosis, migration, EMT, and resistance to tumor cell therapies. These effects are achieved through various mechanisms, such as the lncRNA-miRNA-mRNA ceRNA network, Wnt/β-catenin, JAK/STAT, FAK pathways, and interactions with HIF-1α. In conclusion, LINC00518 participates in the pathogenesis of human diseases and has significant clinical potential as a diagnostic or prognostic biomarker and therapeutic target.

However, these studies have some limitations. An initial concern is that the function of LINC00518 in normal tissues has not been fully investigated. Existing research primarily focuses on its role in cancer, with limited exploration of its expression and biological function in normal tissues. This remains an important area for further investigation. Moreover, although lncRNAs can be tracked as biomarkers in plasma, research on LINC00518 in plasma concerning tumors is still lacking. In addition, in cancers such as cervical cancer, head and neck squamous cell carcinoma, colon cancer, prostate cancer, and multiple myeloma, only one or two studies describe the role of LINC00518 in their development. The paucity of multiple experiments may lead to misunderstandings and controversial results. Furthermore, the sponge mechanism of LINC00518 for miR-216–5p is significant in prostate cancer and non-small cell lung cancer. Further research on LINC00518 and miR-216–5p as potential new targets for precise tumor treatment is warranted. Finally, the influence of polymorphisms within the LINC00518 gene on cancer risk has not been studied. These polymorphisms could affect the biological mechanisms of this lncRNA, thereby impacting the functional role of LINC00518.

In summary, more animal experiments and clinical studies are needed in the future. So far, LINC00518 has been studied predominantly in melanoma, with limited experiments in other tumors. Whether LINC00518 plays a significant role in the pathogenesis of human cancers requires solid support from a broader range of disease types.

## Author contributions

QY: Conceptualization, Data curation, Formal analysis, Investigation, Project administration, Software, Visualization, Writing – original draft. GZ: Conceptualization, Data curation, Project administration, Visualization, Writing – review & editing. WZ: Conceptualization, Formal analysis, Methodology, Project administration, Writing – review & editing. JW: Conceptualization, Data curation, Visualization, Writing – review & editing. XO: Conceptualization, Methodology, Writing – review & editing. KY: Visualization, Writing – review & editing. JZ: Data curation, Funding acquisition, Methodology, Resources, Supervision, Validation, Writing – review & editing.
